# Reply to “Hyperoxemia in postsurgical sepsis/septic shock patients is associated with reduced mortality”

**DOI:** 10.1186/s13054-022-04045-6

**Published:** 2022-06-13

**Authors:** Marta Martín-Fernández, María Heredia-Rodríguez, Eduardo Tamayo

**Affiliations:** 1 Department of Medicine, Toxicology and Dermatology, University of Valladolid, Valladolid, Spain; 2 BioCritic, Group for Biomedical Research in Critical Care Medicine, Valladolid, Spain; 3 Centro de Investigación Biomédica en Red de Enfermedades Infecciosas (CIBERINFEC), Instituto de Salud Carlos III, Madrid, Spain; 4 Department of Anaesthesiology and Critical Care, Hospital Clínico Universitario de Salamanca, Salamanca, Spain; 5 Department of Anaesthesiology and Critical Care, Hospital Clínico Universitario de Valladolid, Avda Ramón Y Cajal 3, 47003 Valladolid, Spain; 6 Department of Surgery, University of Valladolid, Valladolid, Spain

## Dear Editor,

We would like to express our gratitude for the great interest generated by our article [1]. We would kindly like to clarify certain points in order to facilitate and stimulate scientific discussion.

In recent years, there has been a growing interest in clarifying the optimal levels of oxygenation in critically ill patients. Summary data from meta-analyses seem to indicate that hyperoxemia worsens outcome [2]. However, according to a recent systematic review with data from eight trials (4415 participants), no differences have been detected in mortality (OR 0.95, 95% CI 0.74–1.22) between high or low oxygen targets in mechanically ventilated adults. However, the high heterogeneity and the overlapping target ranges limit the validity and clinical relevance of these findings, calling for urgent further research to define optimal oxygen therapy targets [3]. There are controversial findings in patients with septic shock. For example, in the ICU-ROX trial, no differences were found when comparing conservative versus liberal oxygen therapy [4]. In addition, the use of hyperoxic therapy reduced the risk of surgical site infections in colorectal surgery, probably due to the bactericidal activity of neutrophils mediated by oxidative killing, a potent mechanism depending on the production of superoxide radicals from molecular oxygen.

We sought to review our historical series of septic patients focusing on the PaO_2_ values at the time of sepsis diagnosis and within the first 48 h after diagnosis. We observed that patients with PaO_2_ > 100 mmHg had lower 90-day mortality than patients with PaO_2_ ≤ 100 mmHg (25.5% vs. 37.0%, *p* = 0.008). The potential association between PaO_2_ levels and risk of 90-day mortality was further evaluated by using a multivariate logistic regression analysis. Using the variables in Table 1 [1], a univariate regression analysis was performed for 90-day mortality in order to select the confounding factors for the multivariate analysis. Variables showing a *p* value < 0.1 with no collinearity were included as adjustment variables in the multivariate analysis. Then we performed a multivariate binary logistic regression model adjusted for very robust variables such as age, APACHE II > 19, PCT (ng/mL) Ln and chronic renal failure, with PaO2 remaining in the model. Binary logistic regression models are widely accepted for mortality studies when the variable of time is not to be taken into account and, in fact, they have been used in recent studies [5]. The use of a COX regression rather than a binary logistic regression is useful when assessing the influence of time on the model and may be of interest when evaluating mortality in patients with cancer, chronic diseases, etc. For acute processes such as sepsis, what is of interest is the attributed mortality, without any significant contribution being made by the fact that the patient dies on day 5 or day 30. However, what should be considered, as in our case, is whether the patient dies at an early or late stage.

Furthermore, our study only showed an association between mortality and PaO_2_ ≤ 100 mmHg. We thought that this finding is clinically relevant and should be validated in a clinical trial fashion design, as stated in our paper. In fact, this is what we are currently undertaking; we have been drafting a project for several months that will be applied for national competitive calls to conduct a clinical trial with the aim of confirming the hypothesis that we have already proposed in our article in critical care.

## Data Availability

Not applicable.
